# *N*-Acetyltransferase 2 Genotypes among Zulu-Speaking South Africans and Isoniazid and *N*-Acetyl-Isoniazid Pharmacokinetics during Antituberculosis Treatment

**DOI:** 10.1128/AAC.02376-19

**Published:** 2020-03-24

**Authors:** Thuli Mthiyane, James Millard, John Adamson, Yusentha Balakrishna, Cathy Connolly, Andrew Owen, Roxana Rustomjee, Keertan Dheda, Helen McIlleron, Alexander S. Pym

**Affiliations:** a South African Medical Research Council, Durban, South Africa; b Wellcome Trust Liverpool Glasgow Centre for Global Health Research, Liverpool, United Kingdom; c Institute of Infection and Global Health, University of Liverpool, Liverpool, United Kingdom; d Africa Health Research Institute, Durban, South Africa; e South African Medical Research Council, Biostatistics Department, Durban, South Africa; f Department of Molecular and Clinical Pharmacology, University of Liverpool, Liverpool, United Kingdom; g Lung Infection and Immunity Unit, Division of Pulmonology, Department of Medicine, University of Cape Town, Cape Town, South Africa; h Division of Clinical Pharmacology, Department of Medicine, University of Cape Town, Cape Town, South Africa

**Keywords:** acetylation, *N*-acetyltransferase, isoniazid, tuberculosis, HIV, pharmacokinetics, drug metabolism, pharmacogenetics

## Abstract

The distribution of *N*-acetyltransferase 2 gene (*NAT2*) polymorphisms varies considerably among different ethnic groups. Information on *NAT2* single-nucleotide polymorphisms in the South African population is limited. We investigated *NAT2* polymorphisms and their effect on isoniazid pharmacokinetics (PK) in Zulu black HIV-infected South Africans in Durban, South Africa. HIV-infected participants with culture-confirmed pulmonary tuberculosis (TB) were enrolled from two unrelated studies. Participants with culture-confirmed pulmonary TB were genotyped for the *NAT2* polymorphisms 282C>T, 341T>C, 481C>T, 857G>A, 590G>A, and 803A>G using Life Technologies prevalidated TaqMan assays (Life Technologies, Paisley, UK). Participants underwent sampling for determination of plasma isoniazid and *N*-acetyl-isoniazid concentrations. Among the 120 patients, 63/120 (52.5%) were slow metabolizers (NAT2*5/*5), 43/120 (35.8%) had an intermediate metabolism genotype (NAT2*5/12), and 12/120 (11.7%) had a rapid metabolism genotype (NAT2*4/*11, NAT2*11/12, and NAT2*12/12). The *NAT2* alleles evaluated in this study were *4, *5C, *5D, *5E, *5J, *5K, *5KA, *5T, *11A, *12A/12C, and *12M. NAT2*5 was the most frequent allele (70.4%), followed by NAT2*12 (27.9%). Fifty-eight of 60 participants in study 1 had PK results. The median area under the concentration-time curve from 0 to infinity (AUC_0–∞_) was 5.53 (interquartile range [IQR], 3.63 to 9.12 μg h/ml), and the maximum concentration (*C*_max_) was 1.47 μg/ml (IQR, 1.14 to 1.89 μg/ml). Thirty-four of 40 participants in study 2 had both PK results and *NAT2* genotyping results. The median AUC_0–∞_ was 10.76 μg·h/ml (IQR, 8.24 to 28.96 μg·h/ml), and the *C*_max_ was 3.14 μg/ml (IQR, 2.39 to 4.34 μg/ml). Individual polymorphisms were not equally distributed, with some being represented in small numbers. The genotype did not correlate with the phenotype, with those with a rapid acetylator genotype showing higher AUC_0–∞_ values than those with a slow acetylator genotype, but the difference was not significant (*P* = 0.43). There was a high prevalence of slow acetylator genotypes, followed by intermediate and then rapid acetylator genotypes. The poor concordance between genotype and phenotype suggests that other factors or genetic loci influence isoniazid metabolism, and these warrant further investigation in this population.

## Abstract (precis)

The distribution of *N*-acetyltransferase 2 gene (*NAT2*) polymorphisms varies considerably among different ethnic groups. Information on *NAT2* single-nucleotide polymorphisms in the South African population is limited. We investigated *NAT2* polymorphisms and their effect on isoniazid pharmacokinetics (PK) in Zulu black HIV-infected South Africans in Durban, South Africa. HIV-infected participants with culture-confirmed pulmonary tuberculosis (TB) were enrolled from two unrelated studies.

## TEXT

Tuberculosis (TB) remains a leading cause of global morbidity and mortality, with approximately 10 million cases and 1.5 million deaths occurring in 2018 (1). South Africa is a high-TB-burden country, with an estimated 301,000 cases in 2018. The so-called short-course treatment regimen recommended in international guidelines, consisting of 6 months of rifampin and isoniazid (INH), supplemented by pyrazinamide and ethambutol in the first 2 months, has remained largely unchanged for several decades. While this regimen can achieve high relapse-free cure rates, a range of host and mycobacterial factors can influence treatment outcomes. There is increasing evidence that interindividual variability in the pharmacokinetics (PK) of drugs within this regimen lead to heterogeneity in clinical outcomes (2, 3).

Pharmacogenomics (PG) describe one cause of PK variability due to polymorphisms in drug-metabolizing enzymes (DME) and transporters. During anti-TB treatment, isoniazid is the paradigmatic case. Isoniazid is acetylated to its major metabolite, *N*-acetyl-isoniazid (AcINH), by the action of hepatic *N*-acetyltransferase 2 (encoded by the *NAT2* gene). AcINH is subsequently rapidly hydrolyzed to acetyl-hydrazine, which is also acetylated to diacetyl-hydrazine, by the action of *NAT2* (4). Accumulated acetyl-hydrazine can be oxidized to form other, potentially hepatotoxic metabolites (4–6). Moreover, the accumulated isoniazid can be metabolized by an alternative pathway, in which it is first hydrolyzed to hydrazine, which has also been implicated in liver injury, before acetylation to acetyl-hydrazine, which is, again, by *NAT2* (4, 7). Hence, the activity of *NAT2* both dictates the metabolism of isoniazid and determines the availability of potentially hepatoxic hydrazine and acetyl-hydrazine metabolites. Within the 870-bp *NAT2* gene, a number of low-activity single-nucleotide polymorphisms (SNPs) have been characterized. The *NAT2* genotype has been shown to determine the rate of acetylation by *NAT2* in several populations (8). Individuals homozygous for the wild-type alleles are characterized as rapid acetylators (RAs), those homozygous for low-activity SNPs are characterized as slow acetylators (SAs), and heterozygotes are characterized as intermediate acetylators (IAs) (9–13). SAs have a higher incidence of side effects, particularly drug-induced hepatitis, during anti-TB therapy, presumably due to higher levels of hepatoxic metabolites (14–20). Among the first-line anti-TB drugs, isoniazid has the greatest early bactericidal activity (EBA), and isoniazid PK parameters have been associated with the rates of cure, sterilization, and acquired drug resistance (3, 21–27). A link between rapid acetylation and an increased risk of poor treatment outcomes has been reported (28, 29).

The *NAT2* genotype is known to differ among ethnic groups, with approximately 40 to 70% of Caucasians, Indians, and African Americans but only about 10% of Asian populations being characterized as SAs (30–42). The *NAT2* genotype is not well characterized in the communities where TB is most prevalent, particularly in sub-Saharan Africa. South Africa has several black ethnic groups, and few have been studied (43–45). Bach et al. characterized 40% of a Zulu population to be phenotypically slow acetylators, but these findings have not been replicated or informed by genotypic analysis (44). Moreover, South Africa has a high prevalence of individuals infected with HIV, and discordant relationships between the *NAT2* genotype and the isoniazid acetylator phenotype have been described among individuals living with HIV in other settings (46, 47).

We therefore characterized the relationship between *NAT2* genotype, isoniazid and AcINH PK, and hepatotoxicity in a cohort of individuals with TB-HIV coinfection in Durban, KwaZulu-Natal, South Africa.

## RESULTS

### Participant characteristics.

One hundred twenty-two individuals living with HIV and participating in two PK studies were included in the study. Eighty participants in study 1 were included in the *NAT2* genotyping analysis and 60 participants in study 1 were included in the PK analysis (with 58 individuals having both PK and genotype data), while 40 participants in study 2 were included in the PK analysis and 40 participants in study 2 were included in *NAT2* genotyping analysis (with 34 individuals having both PK and genotype data). Key characteristics are outlined in Table 1. Participants in study 1 included 60 with pulmonary TB and HIV coinfection, 40 with CD4 counts of >200 cells/mm^3^, and 20 with CD4 counts of <200 cells/mm^3^, as well as 20 participants living with HIV and without TB (who contributed only genotype data). All 40 participants in study 2 had TB and HIV coinfection and had CD4 counts of 200 cells/mm^3^ or below. In the combined studies, 66.7% of participants had CD4 counts of <200 cells/mm^3^ and 33.3% had CD4 counts of >200 cells/mm^3^. The median age was 33.1 years (interquartile range [IQR], 18 to 53 years). Only 15 (12.5%) patients had a body mass index (BMI) of <18.86 kg/m^2^.

**TABLE 1:** Demographic characteristics

Characteristic	Value for participants in:		
	Study 1 (*n* = 80)	Study 2 (*n* = 40)	Overall (*n* = 120)
Demographics			
Median (IQR) age (yr)	33 (18–48)	33.6 (24–53)	33.1 (18–53)
No. (%) of male participants	36 (45)	24 (60.0)	60 (50)
No. (%) of participants of Zulu ethnicity	80 (100)	40 (100)	120 (100)
			
Mean (SD) wt (kg)	58.7 (11.9)	58.9 (9.7)	58.7 (11.2)
Mean (SD) BMI	23.0 (5.2)	23.1 (3.9)	23.1 (4.8)
No. (%) of participants with BMI of <18.5	13 (16.3)	2 (5.0)	15 (12.5)
			
Median (range) CD4 count (no. of cells/mm^3^)	210.5 (10–500)	128 (61–199)	161 (10–500)
No. (%) of participants with CD4 count of <200 cells/mm^3^	40 (50)	40 (100)	80 (66.7)

### *NAT2* genotype and deduced phenotype.

One hundred twenty participants (80 from study 1 and 40 from study 2) were genotyped. The haplotype assignment and deduced acetylator phenotype for each diplotype are shown in Table 2. Allele and haplotype frequencies and deduced phenotypes are outlined in Tables 3 to 5. We identified 12 different alleles in the population. The most common allelic group was NAT2*5 (70.4%), followed by NAT2*12 (27.9%). In the NAT2*5 group, NAT2*5C (21.3%), NAT2*5J (17.5%), NAT2*5D (14.6%), and NAT2*5K (10.4%) were the most common. The NAT2*12 group was predominantly NAT2*12C. The proportions of individuals with each of the deduced phenotypes was 11.7% rapid, 35.8% intermediate, and 52.5% slow acetylators (Table 5).

**TABLE 2:** *NAT2* diplotypes and genotypes and deduced phenotype in the study group

Observed diplotype^*a*^	No. of mutations	*NAT2* genotype	Phenotype
–20000	1	*5D/5K*	Slow
000020	1	*12A/12A*	Rapid
001000	1	*4/11A*	Rapid
001020	6	*12A/12C*	Rapid
002010	2	*11A/12C*	Rapid
002020	4	*12C/12C*	Rapid
01–020	1	*5C/12C*	Intermediate
010010	1	*5D/12A*	Intermediate
010020	2	*5C/12A*	Intermediate
010110	2	*5E/12A*	Intermediate
011010	3	*5D/12C*	Intermediate
011020	15	*5C/12C*	Intermediate
011110	3	*5E/12C*	Intermediate
0200-0	1	*5C/5D*	Slow
020000	1	*5D/5D*	Slow
020010	10	*5C/5D*	Slow
020020	3	*5C/5C*	Slow
020100	3	*5D/5E*	Slow
020110	1	*5C/5E*	Slow
110010	1	*5K/12A*	Intermediate
110110	1	*5K/12C*	Intermediate
111010	5	*5K/12C*	Intermediate
111020	1	*5T/12C*	Intermediate
111110	6	*5J/12C*	Intermediate
120000	7	*5D/5K*	Slow
120010	5	*5C/5K*	Slow
120011	1	*5C/5KA*	Slow
120020	1	*5C/5T*	Slow
120100	7	*5D/5J*	Slow
120110	8	*5C/5J*	Slow
120200	1	*5E/5J*	Slow
211020	1	*5T/12M*	Intermediate
211110	1	*5J/12M*	Intermediate
220001	1	*5K/5KA*	Slow
220100	4	*5J/5K*	Slow
220110	1	*5J/5T*	Slow
2202–0	1	*5J/5J*	Slow
220200	6	*5J/5J*	Slow

a Observed diplotypes are shown as the number of mutations identified in each individual for each SNP. 0, wild type; 1, heterozygous; 2, homozygous; –, blank. The SNP order is positions 282, 341, 481, 590, 803, and 857.

**TABLE 3:** Frequency of *NAT2* alleles in the study group

Polymorphism	No. of participants	% of participants
NAT2*4	1	0.4
NAT2*5	169	70.4
NAT2*11	3	1.3
NAT2*12	67	27.9
		
Total	240	100

**TABLE 4:** Frequency of *NAT2* haplotypes

Haplotype	No. of participants	% of participants
NAT2*4	1	0.4
NAT2*5C	51	21.3
NAT2*5D	35	14.6
NAT2*5E	10	4.2
NAT2*5J	42	17.5
NAT2*5K	25	10.4
NAT2*5KA	2	0.8
NAT2*5T	4	1.7
NAT2*11A	3	1.3
NAT2*12A	14	5.8
NAT2*12C	51	21.2
NAT2*12M	2	0.8
		
Total	240	100

**TABLE 5:** Frequency distribution of *NAT2* genotypes and deduced phenotype in the study group

Genotype	No. of participants	% of participants	Acetylator status
NAT2*4/*11	1	0.8	Rapid
NAT2*12/*12	11	9.2	Rapid
NAT2*11/*12	2	1.7	Rapid
NAT2*5/*12	43	35.8	Intermediate
NAT2*5/*5	63	52.5	Slow
			
Total	120	100	

### Isoniazid and *N*-acetyl-isoniazid PK.

As described above, to assess the sample integrity for study 1, we compared the area under the concentration-time curve from 0 h to infinity (AUC_0–∞_) values from the current analysis with those previously reported for the same samples analyzed in 2010. The median AUC_0–∞_ was 5.53 μg·h/ml (IQR, 3.63 to 9.12 μg·h/ml), when the samples were processed at the University of Cape Town (UCT) in 2009, and 5.70 μg·h/ml (IQR, 3.85 to 7.94 μg·h/ml), when the samples were processed at the Africa Health Research Institute (AHRI) laboratory in 2014, suggesting that the integrity of the samples was maintained for isoniazid but could not be confirmed for AcINH.

**FIG 1:**
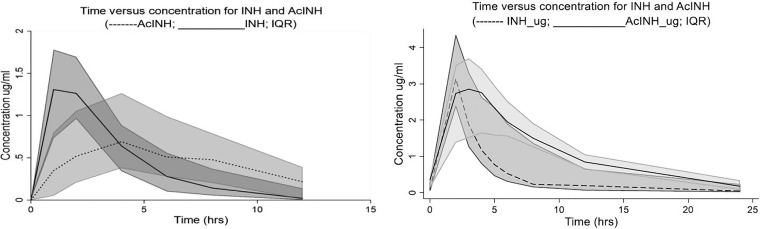
(Left) Study 1 median INH and AcINH concentrations over time for INH and AcINH for 58 patients. (Right) Study 2 median INH and AcINH concentrations over time for INH and AcINH for 34 patients. The shaded areas indicate the IQR.

The participants in study 1 showed rapid absorption, with a median isoniazid time to maximum concentration (*T*_max_) of 1 h (IQR, 1 to 2 h). Isoniazid exposure was variable among individuals, with the median maximum concentration (*C*_max_) being 1.47 μg/ml (IQR, 1.14 to 1.85 μg/ml) and the median AUC_0–∞_ being 5.53 μg·h/ml (IQR, 3.63 to 9.12 μg·h/ml) (Table 6). The median elimination half-life was relatively slow at 2.27 h (IQR, 1.69 to 3.56 h). We compared these isoniazid PK measures to published targets; 98.28% (57/58) of the participants failed to attain the minimum 2-h plasma concentration target of 3 μg/ml (48). PK parameters by genotype are shown in Table 7. Unexpectedly, the median half-life was the slowest, the apparent oral clearance was the lowest, and AUC_0–∞_ was the highest among genotypically rapid acetylators, with the reverse being true for genotypically slow acetylators, although none of these differences was statistically significant. Similarly, there were no statistically significant differences by genotype for the AcINH *C*_max_, elimination half-life, or AUC_0–∞_. The median isoniazid and AcINH time-concentration curves are given in Fig. 1, left.

**TABLE 6:** Overall isoniazid and *N*-acetyl-isoniazid PK^*a*^

Parameter	Value from:			
	Study 1 (*n* = 58)		Study 2 (*n* = 34)	
	Isoniazid	*N*-Acetyl-isoniazid	Isoniazid	*N*-Acetyl-isoniazid
AUC_0–∞_ (μg·h/ml)	5.53 (3.63–9.12)	5.49 (3.18–9.26)	10.76 (8.24–28.96)	27.67 (23.20–34.67)
*C*_max_ (μg/ml)	1.47 (1.14–1.85)	0.90 (0.46–1.40)	3.14 (2.39–4.34)	2.91 (1.73–3.70)
*T*_max_ (h)	1 (1–2)	4 (2–6)	2 (2–2)	3 (3–4)
CL/*F* (liters/h)	47.64 (35.36–74.11)	NA	27.34 (10.83–32.00)	NA
*t*_1/2_ (h)	2.27 (1.69–3.56)	4.28 (3.29–5.79)	2.62 (2.26–4.07)	5.89 (5.04–8.21)

a All values are medians (interquartile ranges). AUC_0–∞_, area under the concentration-time curve from 0 h to infinity; *C*_max_, maximum concentration; *T*_max_, time to maximum concentration; CL/*F*, clearance; *t*_1/2_, elimination half-life; NA, not applicable.

**TABLE 7:** Study 1 PK parameters by genotype^*a*^

Parameter	Value for participants with the indicated acetylator phenotype receiving:					
	Isoniazid			*N*-Acetyl-isoniazid		
	Slow (*n* = 33)	Intermediate (*n* = 12)	Rapid (*n* = 13)	Slow (*n* = 33)	Intermediate (*n* = 12)	Rapid (*n* = 13)
AUC_0–∞_ (μg·h/ml)	5.34 (3.44–7.93)	6.04 (4.27–7.53)	7.56 (5.99–9.60)	5.71 (4.19–11.01)	7.34 (3.15–10.9)	2.81 (0.55–5.06)
*C*_max_ (μg/ml)	1.47 (0.97–1.89)	1.54 (1.25–1.76)	1.42 (1.20–2.05)	0.94 (0.63–1.68)	1.07 (0.49–1.70)	0.38 (0.09–0.90)
*T*_max_ (h)	1 (1–2)	1 (1–2)	2 (2–2)	4 (2–4)	4 (4–7)	6 (4–6)
CL/*F* (liters/h)	57.05 (37.84–103.56)	43.53 (32.05–64.33)	37.75 (31.27–47.92)	NA	NA	NA
*t*_1/2_ (h)	1.87 (1.52–3.07)	2.27 (1.78–3.6)	4.00 (2.98–4.73)	4.36 (3.43–5.25)	4.09 (3.13–5.49)	5.42 (3.58–7.48)

a Data are for 58 participants. All values are medians (interquartile ranges). AUC_0–∞_, area under the concentration-time curve from 0 h to infinity; *C*_max_, maximum concentration; *T*_max_, time to maximum concentration; CL/*F*, clearance; *t*_1/2_, elimination half-life; NA, not applicable.

Absorption was rapid in the participants in study 2, with the median INH *T*_max_ being 2 h. INH exposure was also variable among the individuals, with the median *C*_max_ being 3.14 μg/ml (IQR, 2.39 to 4.34 μg/ml) and the median AUC_0–∞_ being 10.76 μg·h/ml (IQR, 8.24 to 28.96 μg·h/ml). The median elimination half-life was 2.62 (IQR, 2.26 to 4.07) (Table 6). Again, we compared these INH PK measures to published PK targets; 47.5% (19/40) of the participants failed to attain the minimum 2-h plasma concentration target of 3 μg/ml. PK parameters by genotype are shown in Table 8. For both isoniazid and AcINH and across the PK parameters *C*_max_, AUC_0–∞_, and elimination half-life, variability (both range and IQR) was increased among those genotyped as SAs. Again, however, there were no statistically significant differences between these PK parameters by genotype. Median isoniazid and AcINH time-concentration curves are given in Fig. 1, right.

**TABLE 8:** Study 2 PK parameters by genotype^*a*^

Parameter	Value for participants with the indicated acetylator phenotype receiving:					
	Isoniazid			*N*-Acetyl-isoniazid		
	Slow (*n* = 22)	Intermediate (*n* = 11)	Rapid (*n* = 1)	Slow (*n* = 22)	Intermediate (*n* = 11)	Rapid (*n* = 1)
AUC_0–∞_ (μg·h/ml)	10.76 (9.73–31.21)	9.09 (7.3–18.75)	26.99	26.04 (22.99–32.76)	6.28 (5.25–10.01)	28.53
*C*_max_ (μg/ml)	3.47 (2.49–4.49)	2.96 (2.33–4.02)	3.94	2.85 (1.52–3.68)	3.28 (2.53–4.01)	1.91
*T*_max_ (h)	2 (2–2)	2 (2–2)	2	3 (3–4)	3 (3–3)	4
CL/*F* (liters/h)	27.87 (9.66–30.83)	33.33 (16.01–41.17)	11.12	NA	NA	NA
*t*_1/2_ (h)	2.64 (2.26–4.08)	2.38 (2.16–2.58)	5.26	5.81 (4.9–7.25)	6.28 (5.25–10.01)	10.97

a Data are for 34 participants. All values are medians (interquartile ranges). AUC_0–∞_, area under the concentration-time curve from 0 h to infinity; *C*_max_, maximum concentration; *T*_max_, time to maximum concentration; CL/*F*, clearance; *t*_1/2_, elimination half-life; NA, not applicable.

**FIG 2:**
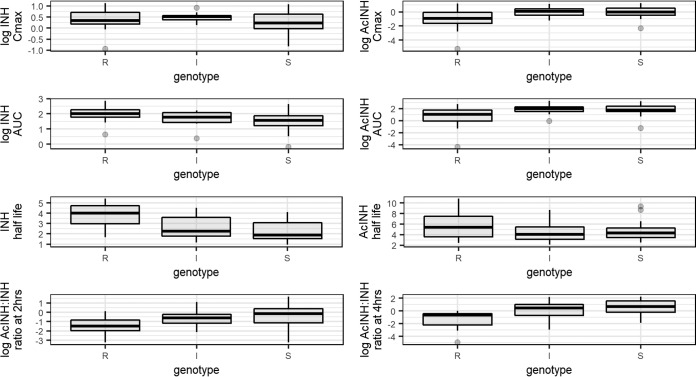
Box plots for study 1, representing the medians (solid lines), interquartile ranges (boxes), and ranges (whiskers) for the pharmacokinetic parameters log maximum concentration (*C*_max_), log area under the concentration-time curve from 0 h to infinity (AUC_0–∞_), and half-life for isoniazid (INH) and *N*-acetyl-isoniazid (AcINH) stratified by acetylator status and log AcINH concentration to log INH concentration ratio at 2 and 4 h stratified by acetylator genotype. R, I, and S, rapid, intermediate, and slow acetylator genotypes, respectively.

**FIG 3:**
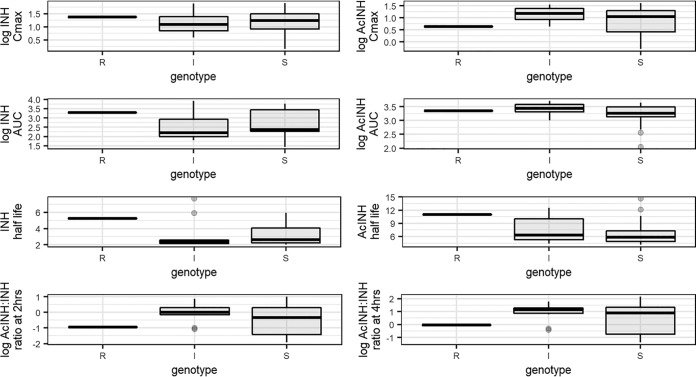
Box plots for study 2, representing the medians (solid lines), interquartile ranges (boxes), and ranges (whiskers) for the pharmacokinetic parameters log maximum concentration (*C*_max_), log area under the concentration-time curve from 0 h to infinity (AUC_0–∞_), and half-life for isoniazid (INH) and *N*-acetyl-isoniazid (AcINH) stratified by acetylator status and log AcINH concentration to log INH concentration ratio at 2 and 4 h stratified by acetylator genotype. R, I, and S, rapid, intermediate, and slow acetylator genotypes, respectively.

For both studies, we calculated the log AcINH concentration/log isoniazid concentration ratio as a measure of acetylation at 2 and 4 h postdose and analyzed this ratio by genotype (Fig. 2 and 3). In both studies we saw no statistically significant difference in ratios between genotypes at either 2 or 4 h. In study 2, we again saw increased variability in this metric among those genotyped as SAs.

### Hepatic adverse events.

There were no grade 3 and 4 hepatic adverse events in study 1, and only 1 grade 4 hepatic event was reported from the only participant with a rapid acetylator genotype in study 2. Although there were more grade 1 hepatic adverse events among the slow acetylator genotype participants than among the participants with the other genotypes, as shown in Table 9, the difference was not statistically significant between genotypes (*P* = 0.203 in study 1 and *P* = 0.276 in study 2).

**TABLE 9:** Participants with any hepatic adverse events^*a*^

Adverse event grade	No. (%) of participants with the indicated acetylator phenotype							
	Study 1				Study 2			
	Rapid	Intermediate	Slow	Total	Rapid	Intermediate	Slow	Total
1	7	9	25	41	0	5	10	15
2	2	0	0	2	0	1	1	2
3	0	0	0	0	0	2	1	3
4	0	0	0	0	1	0	0	1
								
Total	9 (20.9)	9 (20.9)	25 (61)	43 (100)	1 (4.8)	8 (30.1)	12 (57.1)	21 (100)

a Hepatic adverse events from the two studies included a combination of elevated aspartate aminotransferase (AST), alanine aminotransferase (ALT), alkaline phosphatase (ALP), gamma-glutamyltransferase, and total bilirubin levels.

## DISCUSSION

We investigated the *NAT2* genotype and the PKs of isoniazid and AcINH in black Zulu South Africans living with HIV from Durban and surrounding areas. We found that most individuals were of the SA (52.5%) or IA (35.8%) genotype, with only a small number being of the RA genotype (11.7%). The proportions of the deduced acetylator phenotypes in our population were broadly similar to those in other African and Caucasian populations (36, 43, 49, 50) but differed from those previously reported from within other black ethnic groups within southern Africa. For example, Werely found that IA genotypes dominated in the Xhosa cohort, with individuals of the SA genotype comprising only 30% of the cohort (45). Our results were comparable to those of a recent study by Naidoo et al. in patients from the same geographic area, who reported 34% SAs, 43% IAs, and 18% RAs (51).

There was a high prevalence of the NAT2*5 allelic group, accounting for the slow acetylator genotype, in our population. In well-studied Caucasian and Asian populations, four variants, NAT2*4 (wild type, rapid acetylators) and NAT2*5B, NAT2*6A, and NAT2*7B (all slow acetylators), accounted for most *NAT2* alleles. In Asian populations, there are generally a higher proportion of wild-type NAT2*4 alleles and few NAT2*5B alleles, and this difference largely accounts for the much lower prevalence of RAs in non-Asian populations. Consistent with other studies in sub-Saharan African populations, the wild-type NAT*4 allele was far less prevalent and variant alleles were far more diverse in our study. In our population, the NAT2*5B allele was relatively rare in comparison to its occurrence in two studies in the black population from the Western Cape and North West Provinces in South Africa (45, 52). However, in contrast to these South African populations, there were a diversity of other NAT2*5 alleles, including a much higher prevalence of the rare NAT2*5J allele (17.5%) and the poorly characterized NAT2*5K allele (10.4%). The NAT2*6A and NAT2*7B alleles, common in Caucasian and Asian populations, were not seen in our cohort. In Caucasian and Asian populations, rapid acetylator NAT2*12 alleles are rarely seen, whereas in populations in sub-Saharan Africa, the NAT2*12A allele is reported at much higher frequencies (35). In our study, the NAT2*12A allele did indeed comprise 5.8% of alleles seen, but we saw a much higher frequency of the NAT2*12C allele (21.2%), in contrast to its frequency in other southern African cohorts (10, 45, 52, 53).

The isoniazid *C*_max_ and AUC_0–∞_ demonstrated considerable variability between individuals in both studies, and almost all participants in study 1 and almost half of the participants in study 2 had a *C*_max_ below the lower limit of the target range (48). Low isoniazid concentrations during anti-TB treatment are concerning because it has been postulated that they may lead to poorer treatment outcomes or the generation of isoniazid resistance, the likely first step in the evolution of multidrug-resistant TB (MDR TB). However, the evidence for either of these concerns is mixed, and in this setting, the prevalence of INH monoresistance is relatively low.

There was a marked difference in PK measures between the two studies analyzed, with study 1 having much lower measures than study 2. There are several reasons that could have contributed to this difference. The difference in isoniazid dosing could explain the lower PK measures, where study 1 used the fixed-dose combination (FDC) dosing per World Health Organization (WHO)-recommended weight bands, leading to almost half the participants receiving doses of <300 mg, as previously reported (54). All participants in study 2 received 300-mg doses of isoniazid irrespective of weight. Although the samples from study 1 did not appear to deteriorate during the 5 years between the first analysis and subsequent analyses for this study, the possibility of differences in processing and storage between the studies cannot be excluded. Figure 3 shows the PK measures for INH and AcINH at different time points. Based on those findings, the acetylator phenotype of the study participants was generally more intermediate/rapid than what the predominant slow genotype suggests, which is in contrast to other studies reporting that HIV-infected patients have a tendency toward a slow acetylator phenotype (55).

We identified no statistically significant difference by *NAT2* genotype in a variety of PK measures; hence, in this cohort we found a poor correlation between the *NAT2* genotype and the phenotypic acetylation of isoniazid. Previous studies in other populations have shown a good correlation between the *NAT2* genotype and isoniazid PK, suggesting that *NAT2* genotyping could be used as a parsimonious way to risk stratify patients and personalize the dosing of isoniazid in an attempt to maximize efficacy while minimizing toxicity. There are significant practical difficulties to implementing these approaches in this setting, but our data suggest that in this population, *NAT2* genotyping will not be helpful in guiding anti-TB therapy. A lack of concordance between genotypic and phenotypic measures of INH acetylation has previously been reported in HIV-positive cohorts (56, 57). It is likely that in this cohort, as in others, other, nongenetic factors are more important than or as important as the *NAT2* genotype. Jones et al. found that infection with HIV or the stage of HIV infection may alter phase I and II drug-metabolizing enzyme (DME) activity in their study of 17 HIV-infected participants at different levels of immunosuppression (58). They found that HIV infection was related to an increase in the variability of these DMEs. While additional pathways, aside from *NAT2* genotype, have been implicated in the hepatotoxicity of isoniazid-containing anti-TB treatment regimens, it is not clear that these pathways alter isoniazid PK and thus could account for the lack of genotypic and phenotypic concordance in this study.

Although there were more hepatic adverse events among the SAs, there was no statistically significant association between genotype and hepatotoxicity in the two studies, with only 1 patient who was an RA having a grade 4 hepatic adverse event and 2 others who were IAs having grade 3 hepatic adverse events.

In our study, the participants received pyridoxine and co-trimoxazole with the antituberculosis therapy (ATT) in study 2 but not in study 1, as we used the samples collected on day 1 for this analysis when only ATT was given. As both INH and sulfamethoxazole are inhibitors of CYP2C9, this could be one of the reasons for the variations noted. INH also inhibits CYP3A4, which is induced by rifampin, but this interaction has not proven significant except when it relates to hepatotoxicity (59, 60). That the combination of INH and rifampin leads to an increased risk of hepatotoxicity has been reported in other studies. In our study 2, isoniazid was given with rifabutin, which is a less potent hepatic enzyme inducer than rifampin, which therefore should have less interaction with INH (61). Considering the limited effect on hepatotoxicity, the effect of CYP2E1 was not evident in our study. We cannot confirm or exclude the possibility of an effect of these CYP450 enzymes on INH metabolism in these participants.

In our study, the samples were stored at −80°C and the loss of compound due to storage would have been minimal (62), although studies have not reported on the results for plasma samples stored longer than 5 weeks, nor have they reported on sample integrity for the metabolite, AcINH.

### Conclusion.

Among black Zulu TB-HIV-coinfected South African patients, most had a slow or intermediate acetylator *NAT2* genotype. There was a diversity of specific *NAT2* alleles, with the pattern differing from that in previously studied cohorts in other settings. Despite the rarity of rapid acetylator genotypes, INH PK were variable, and a substantial proportion of individuals failed to attain minimum efficacy targets. Importantly, the *NAT2* genotype did not explain the PK variability in this cohort or the low *C*_max_, which suggests that other factors could be influencing isoniazid bioavailability and metabolism, and these require further elucidation.

## MATERIALS AND METHODS

### Participants, study treatment, and sample collection.

Participants from two unrelated PK studies were included (54, 63). Both studies recruited black, Zulu-speaking adults living with HIV from KwaZulu-Natal, South Africa, between March 2007 and April 2010. Study 1 was entitled “Bioavailability of the Fixed Dose Formulation Rifafour Containing Isoniazid, Rifampin Pyrazinamide, Ethambutol and the WHO Recommended First Line Anti-Retroviral Drugs Zidovudine, Lamivudine, Efavirenz Administered to New TB Patients at Different Levels of Immunosuppression.” The results of this study have been previously reported (54). As shown in Table 10, for the purposes of this analysis, we used samples collected on day 1 of the study after an overnight fast, at predose, at 1, 2, 4, 5, 6, 8, and 12 h postdose, with the samples being analyzed for INH and AcINH for 60 participants with microbiologically proven pulmonary TB (by a positive sputum culture or smear) who received a standard first-line TB regimen consisting of an FDC, as described above. The INH dose was 150 mg, 225 mg, 300 mg, and 375 mg per day for participants with weights of 30 to 37 kg, 38 to 54 kg, 55 to 70 kg, and 70 kg and above, respectively, per WHO guidelines (64). Blood from each participant was collected in a PAXgene tube for *NAT2* genotyping. In addition, genotyping was performed on a further 20 participants without TB who were recruited to this study (54).

**TABLE 10:** PK time points and dosing^*a*^

Study	Schedule of PK sampling (day of anti-TB treatment)	Treatment
Study 1	Day 1, with sampling predose and at 1, 2, 4, 6, 8, and 12 h after the dose	Four-drug FDC formulation (EMB, RMP, INH, and PZA at 275, 150, 75, and 400 mg, respectively) dosed daily by weight band^*b*^
Study 2		Enrollment at wk 6 and standard weight band-based treatment with RMP, INH, PZA, and EMB (as in study 1)
		At wk 6 and 7, RMP was replaced with RFB at 300 mg daily
	Day 63 (after 2 wk on continuation phase RBN-INH) with sampling predose and 2, 3, 4, 5, 6, 8, 12, and 24 h after the dose	At wk 8 and 9, RFB at 300 mg and INH at 300 mg

a PK, pharmacokinetics; RMP, rifampin; PZA, pyrazinamide; EMB, ethambutol; FDC, fixed-dose combination; RFB, rifabutin (Mycobutin, Pfizer).

b Participants weighing 30 to 37 kg received 2 tablets, those weighing 38 to 54 kg received 3 tablets, those weighing 55 to 70 kg received 4 tablets, and those weighing >70 kg received 5 tablets.

Study 2, entitled “Pharmacokinetics of Rifabutin Combined with Antiretroviral Therapy in the Treatment of Tuberculosis Patients with HIV Infection in South Africa,” was a randomized controlled trial of two different rifabutin doses coadministered with lopinavir-ritonavir-based antiretroviral therapy (63, 65). The participants initially received 6 weeks of standard intensive-phase treatment, followed by 2 weeks with rifabutin at 300 mg daily, which replaced rifampin. After 2 weeks of the continuation phase, during which the participants received only isoniazid and rifabutin (both at 300 mg daily), PK sampling was carried out. Individuals were fasted overnight, and a standard hospital breakfast was served 2 h after drug ingestion. Sampling was conducted predose and at 2, 3, 4, 5, 6, 8, 12, and 24 h after drug intake, with samples being analyzed for isoniazid and AcINH for 40 participants. *NAT2* genotyping was performed on 40 participants, with 34 participants having both PK sampling and genotyping.

All participants receiving anti-TB treatment in both studies were given pyridoxine for peripheral neuropathy prophylaxis, and patients with CD4 counts below 200 cells/mm^3^ received co-trimoxazole. No participants were on antiretrovirals at the time of PK sampling. Both studies were approved by the South African Medicines Control Council (SAMCC), Biomedical Research Ethics Committee (BREC), of the University of KwaZulu-Natal (study 1, approval number E294/05; study 2, approval number BFC011/07) and the South African Medical Research Council (SAMRC) Ethics Committee. Study 1 was also approved by the WHO Ethics Research Ethics Committee. Written informed consent was obtained from all participants.

### *NAT2* genotyping procedures.

Total genomic DNA was isolated from whole blood using a QIAamp DNA minikit (Qiagen, Crawly, UK) according to the manufacturer’s instructions. Participants were genotyped, using a DNA Engine Chromo4 system (Bio-Rad Laboratories, Hercules, CA) and Opticon Monitor (v.3.1) software (Bio-Rad Laboratories), for 6 *NAT2* SNPs, 282C>T, 341T>C, 481C>T, 857G>A, 590G>A, and 803A>G, using Life Technologies prevalidated probe-based TaqMan assays per the manufacturer’s instructions (Life Technologies, Paisley, UK). Each participant sample was analyzed in duplicate.

### Haplotype assignment and acetylator genotype inference.

Haplotype assignment from probe-based SNP data is poorly described in African populations. We elected to employ an unbiased Phase analysis, which takes the data set as a whole to assign the most likely haplotype for each individual, alongside a probability for this assignment (66, 67). The haplotype for each individual and the acetylator genotype for each haplotype were defined per the guidelines of the *NAT* Gene Nomenclature Committee (http://nat.mbg.duth.gr/Human%20NAT2%20alleles_2013.htm). Individuals with two rapid acetylator alleles were defined as RAs, those with two slow acetylator alleles were defined as SAs, and those with one fast acetylator allele and one slow acetylator allele were defined as IAs.

### Isoniazid and *N*-acetyl-isoniazid PK and phenotype inference.

Blood samples were collected and immediately placed on ice, before centrifugation within 60 min, immediate separation, and storage of plasma at −70°C until analysis. The concentrations of isoniazid, AcINH, and the 6-aminonicotinic acid internal control were quantified using validated high-performance liquid chromatography and tandem mass spectrometry (HPLC-MS/MS). Sample preparation included a protein precipitation step with acetonitrile and subsequent dilution with water. The analytes were chromatographically separated using a Waters Exterra C_18_ column (particle size, 3.5 μm; 50 mm by 2.1 mm) and detected using an AB Sciex 5500 Q-Trap mass spectrometer. All analytes were analyzed isocratically with an acetonitrile–water–0.1% formic acid mobile phase. Isoniazid, AcINH, and the internal standard were analyzed at mass transitions of the precursor ions (*m/z*) of 137.9, 180.1, and 138.7 to the product ions (*m/z*) of 66.0, 78.6, and 50.9, respectively. Chromatographic data acquisition, peak integration, and quantification of analytes were performed using Analyst software (version 1.5.2). We constructed time-concentration curves in the PK package in R for Windows (version 3.5.1). We characterized the isoniazid and AcINH PK parameters maximum concentration (*C*_max_), time to maximum concentration (*T*_max_), area under the concentration-time curve from 0 h to infinity (AUC_0–∞_), apparent oral clearance (CL), and elimination half-life and compared *C*_max_ to published efficacy targets (48). AUC_0–∞_ was calculated using the trapezoid rule, apparent oral clearance was estimated by dose/AUC_0–∞_, and the elimination half-life was estimated by regression analysis of the log concentrations of the terminal exponent of elimination. We analyzed the ratio of log AcINH concentration/log isoniazid concentration at 2 and 4 h to assess the acetylation phenotype.

Sample processing and HPLC-MS were initially conducted in 2010 for study 1. Samples remained in storage and were later moved to a new storage facility before they were shipped to a different laboratory for determination of isoniazid and AcINH concentrations as described above (having previously only had isoniazid concentrations determined). To confirm the integrity of these samples, we compared the isoniazid AUC_0–∞_ from the current analysis with that previously reported for the same samples analyzed in 2010.

### Statistical methods.

All data were entered into EpiData software and transferred to either Stata (version 14) or R for Windows (version 3.5.1) for statistical analysis. Demographic characteristics were presented as frequencies and percentages for categorical variables and as means with standard deviations for continuous variables. Descriptive PK data were described as the median and interquartile ranges. *C*_max_ and AUC_0–∞_ were log transformed prior to comparison between genotypes. PK parameters were compared, by genotype, using the Wilcoxon rank-sum test or the Kruskal-Wallis test.

### Hepatic adverse events.

Hepatic adverse events were defined as elevated alanine transaminase (ALT) and aspartate transaminase (AST) levels, elevated alkaline phosphatase levels, and elevated total bilirubin levels, graded per the Division of AIDS, NIAID, NIH, toxicity table for grading the severity of HIV-positive adult adverse events.

### Data availability.

Data related to this study have been deposited at https://figshare.com/s/8b42c433e1edce625849.
